# The Uncertainties of Bacteriological Diagnosis

**Published:** 1902-10-04

**Authors:** 


### THE UNCERTAINTIES OF BACTERIOLOGICAL DIAGNOSIS.

In discussing the value of bacteriological diagnosis in diphtheria, Professor Sims Woodhead[1] says that during the course of two or three years he personally examined or reviewed the examinations of 27,128 cultivations, of which number 24,933, taken from 12,172 patients, were tabulated. Of these 12,172 cases sent into hospital to be treated for diphtheria, many of them being subjected to repeated bacteriological examination, 8,937, or 73·42 per cent., were found to have diphtheria bacilli in the throat, whilst in 3,235 cases, or 26·58 per cent., no bacilli could be demonstrated. In a certain portion of the cases in which bacilli were not found they were in all probability present, and this being so, he mentions various conditions by which the results of bacteriological examination may be vitiated. On many occasions swabs taken from nurses who have been attending diphtheria cases, but who appeared to be perfectly healthy themselves, have furnished bacilli, and we now know, says Professor Woodhead, that diphtheria bacilli may, under certain conditions, be present in apparently healthy throats, the individual never manifesting a trace of local diphtherial mischief. But we also know that should the general health of the individual be impaired, or should conditions favourable to the development of an attack of catarrh be developed an attack of diphtheria will supervene.

On the other hand, in a number of cases in which the clinical history undoubtedly pointed to an attack of true diphtheria, no diphtheria bacilli could be demonstrated. But many of these were children of tender years from whom it appears to be an exceedingly difficult matter to obtain satisfactory swabs, especially where the disease is confined to the trachea. One need not therefore be astonished at obtaining negative results in such cases. In some cases the diphtheria bacillus appears to maintain its position on the surface of the false membrane for a comparatively short period, and in these cases one may be unable to find the organism in the cultivation. This is especially the case where certain other organisms are present in large numbers. For example it has often happened that in cultures taken from patients *in extremis*, or after death (although there could be no doubt about the diphtheria) no specific bacilli could be found. Other organisms were present in abundance, and it would seem as if the diphtheria bacillus after doing its work had been ousted from its position on the surface of the mucous membrane by the numerous putrefactive and other organisms which as death approaches make their appearance in the mouth. This doubtless explains a certain proportion of the differences that have arisen between the clinical observer and the bacteriologist.

In cases of laryngeal diphtheria again many swabs are unsatisfactory from the very outset, no bacilli being found on them. The necessity for repeated examination, not only in diagnosis but also in connection with the after treatment, is brought out in the fact that although in a certain percentage of cases no diphtheria bacilli were found at first, they were demonstrated at subsequent examinations. In some cases a negative examination was not only preceded but followed by examination in which the bacilli were present, whilst in others a positive diagnosis was both preceded and succeeded by a negative one. Professor Woodhead may well say then, "I am satisfied from all this that a single examination is not sufficient." What is quite evident is that no medical officer of health can, on the strength of a swab examination, safely stand up in court and swear that this is, or that is not, a case of diphtheria — at any rate if the cross-examining lawyer is worth his salt.

[1] Brit. Med. Jour., Sept. 27.

